# The Sphingosine-1-phospate receptor 1 mediates S1P action during cardiac development

**DOI:** 10.1186/1471-213X-11-37

**Published:** 2011-06-13

**Authors:** Ryan R Poulsen, Carolyn M McClaskey, Scott A Rivkees, Christopher C Wendler

**Affiliations:** 1 Section of Developmental Endocrinology and Biology, Yale Child Health Research Center, Department of Pediatrics, Yale University School of Medicine, New Haven, Connecticut 06520, USA

## Abstract

**Background:**

Sphingosine-1-phosophate (S1P) is a biologically active sphingolipid metabolite that influences cellular events including differentiation, proliferation, and migration. S1P acts through five distinct cell surface receptors designated S1P_1-5_R, with S1P_1_R having the highest expression level in the developing heart. S1P_1_R is critical for vascular maturation, with its loss leading to embryonic death by E14.5; however, its function during early cardiac development is not well known. Our previous studies demonstrated that altered S1P levels adversely affects atrioventricular (AV) canal development *in vitro*, with reduced levels leading to cell death and elevated levels inhibiting cell migration and endothelial to mesenchymal cell transformation (EMT).

**Results:**

We determined, by real-time PCR analysis, that S1P_1_R was expressed at least 10-fold higher than other S1P receptors in the developing heart. Immunohistochemical analysis revealed S1P_1_R protein expression in both endothelial and myocardial cells in the developing atrium and ventricle. Using AV canal cultures, we observed that treatment with either FTY720 (an S1P_1,3,4,5_R agonist) or KRP203 (an S1P_1_R-specific agonist) caused similar effects on AV canal cultures as S1P treatment, including induction of cell rounding, inhibition of cell migration, and inhibition of EMT. *In vivo*, morphological analysis of embryonic hearts at E10.5 revealed that S1P_1_R-/- hearts were malformed with reduced myocardial tissue. In addition to reduced myocardial tissue, E12.5 S1P_1_R-/- hearts had disrupted morphology of the heart wall and trabeculae, with thickened and disorganized outer compact layer and reduced fibronectin (FN) deposition compared to S1P_1_R+/+ littermates. The reduced myocardium was accompanied by a decrease in cell proliferation but not an increase in apoptosis.

**Conclusions:**

These data indicate that S1P_1_R is the primary mediator of S1P action in AV canal cultures and that loss of S1P_1_R expression *in vivo *leads to malformed embryonic hearts, in part due to reduced fibronectin expression and reduced cell proliferation.

## Background

Sphingosine-1-phosphate (S1P) is a biologically active lysophospholipid that is involved in cellular differentiation, proliferation, migration, cytoskeletal reorganization, and apoptosis [1,2]. S1P is produced by sphingosine kinase 1 and 2 (SPHK1 and SPHK2) from sphingosine in response to various cellular stimuli, including vascular endothelial growth factor (VEGF), platelet-derived growth factor (PDGF), tumor necrosis factor-α (TNFα), transforming growth factor-beta (TGFβ), epidermal growth factor (EGF) and cytokines [1-5]. After release from cells, S1P acts in an autocrine and paracrine manner through its cell surface receptors to influence cellular processes.

S1P receptors (S1PRs) are G protein-coupled receptors (GPCRs) critical for S1P action; five subtypes have been described S1P_1-5_R (formerly Edg1, Edg5, Edg3, Edg6, and Edg8, respectively)[6,7]. Three S1P receptor subtypes (S1P_1,2,3_R) are expressed in the adult cardiovascular system, each with a unique pattern of expression [8,9]. S1P_1_R is strongly expressed in cardiomyocytes and vascular endothelium [8]. S1P_2_R is the dominate receptor in vascular smooth muscle cells [8]. S1P_3_R is highly expressed in cardiac fibroblasts [8].

In the developing embryo, S1P_1,2,3,4_R expression is detected in the murine heart from embryonic days (E) 8.5-12.5 by reverse transcriptase (RT) PCR [10]. S1P_5_R expression is not detected in the developing heart by either RT-PCR or *in situ *hybridization [10,11]. S1P_1_R is the only S1P receptor detected in the heart from E8.5 to E12.5 by *in situ *hybridization [11]. S1P_1_R is exclusively expressed in the heart at E8.5-E9.5 and is strongly expressed in the heart and developing vasculature throughout the embryo from E10.5-12.5, as well as other tissues including branchial arches, limb buds, and brain [11].

S1P action is implicated in the regulation of numerous cardiovascular processes including angiogenesis, vascular permeability, arteriogenesis, cardiac function, vascular development, and vascular tone [9,12,13]. In fish, deletion of S1P_2_Rs (*miles apart*) results in cardia bifida, which is caused by a failure of the myocardial precursor cells to migrate to the ventral midline of the embryo and fuse to form the heart tube [14].

In mice, deletion of S1P_1_R causes embryonic death between E13.5 and E14.5 due to defects in vascular maturation [15]. In S1P_1_R-/- embryos, the failure of vascular smooth muscle cells (VSMC) to surround and support the developing vasculature results in massive hemorrhaging [15,16]. The bleeding observed in S1P_1_R-/- embryos results from a loss of receptor expression specifically in the endothelium [15,16]. The loss of S1P_2_R or S1P_3_R individually have no adverse affects on cardiovascular development in mice, and null animals are viable [17]. However, loss of both S1P_2_R and S1P_3_R leads to reduced viability after E13.5, possibly due to abnormal endothelium formation in microvessels [18-20]. Although an important role for S1P in vascular development has been observed [15], our understanding of the role of S1P during cardiac development is limited.

Disruptions in cardiac cushion tissue development can lead to septation and valve defects in the heart, which are among the most common congenital heart malformations observed in humans [21,22]. A major component of cardiac cushion development is the transformation of endothelial cells into mesenchymal cells that invade the cushion tissue and contribute to the formation of mature heart valves [23,24]. Some signaling molecules, including TGFβ and VEGF, that are involved in promoting and inhibiting endothelial to mesenchymal cell transformation (EMT) in the heart can also regulate S1P production by influencing SPHK [4,5,25-28].

We demonstrated that altered S1P signaling disrupts cell morphology and cell survival in cardiac cushion tissue [10]. Elevated S1P levels lead to changes in the actin cytoskeleton and cell rounding [10]. In addition, S1P inhibits cell migration and prevents endothelial to mesenchymal cell transformation (EMT) in atrioventricular (AV) canal cushions [10]. In contrast, reducing S1P synthesis by treating with N, N-dimethylsphingosine (DMS), an inhibitor of SPHK, causes apoptosis of myocardial and endocardial cells in AV canal cushions [10].

We now identify S1P_1_Rs as the primary mediators of S1P action in cardiac cushion tissue, and demonstrate that loss of S1P_1_Rs disrupts cardiac development, in part by reducing cell proliferation and reducing FN expression in the heart.

## Results and Discussion

### S1P_1_R is the most highly expressed S1P receptor in the developing heart

**Figure 1:**
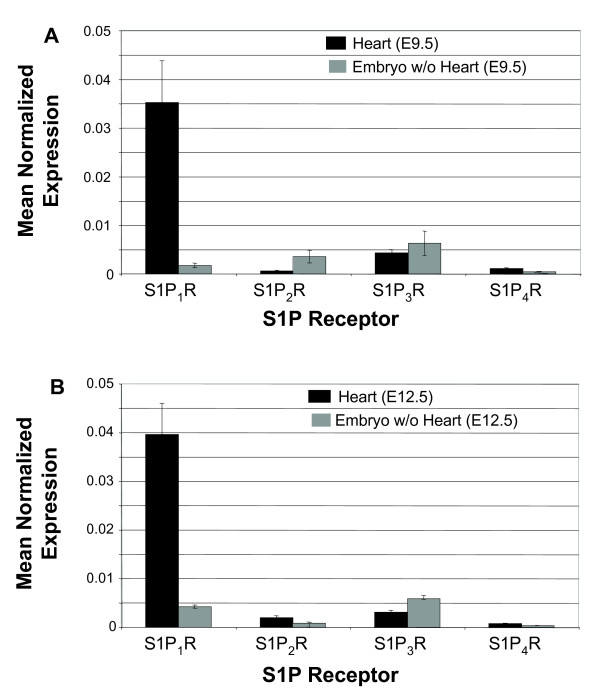
**S1P_1_R is the highest expressed S1P receptor in the developing heart**. Real-time PCR analysis was performed on RNA samples isolated from either hearts only or embryos with hearts removed at (A) E9.5 and (B) E12.5. S1P receptor gene expression was compared to β-actin expression to determine the mean normalized expression. Each analysis was performed in triplicate.

To assess relative expression levels of S1P receptors, we performed quantitative real-time PCR analysis with RNA isolated from embryonic hearts at E9.5, the beginning of cardiac cushion development, and at E12.5, a late stage of cardiac cushion development. Please note that specific radiolabeled ligands are not available to directly characterize receptor binding site expression. Real-time PCR revealed that S1P_1_R is the predominate S1P receptor expressed in E9.5 and E12.5 embryonic hearts and that S1P_2_R, S1P_3_R, and S1P_4_R are expressed at very low levels compared to S1P_1_R (Figure 1). Using mean normalized expression methods to analyze real-time PCR results, it was observed that S1P_1_R was expressed at greater than 10-fold higher levels than other S1P receptor subtypes in the developing heart, and that S1P_1_R gene expression in the rest of the embryo was low compared to the heart at these early embryonic stages (Figure 1).

**Figure 2:**
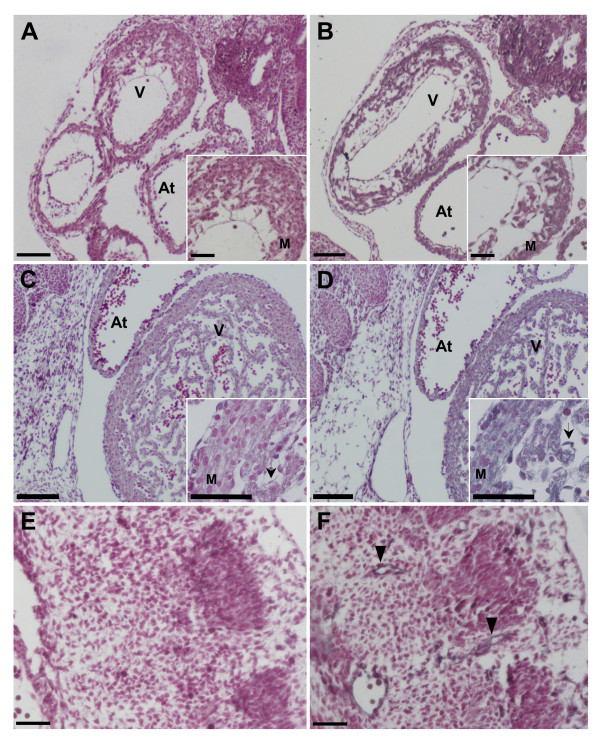
**S1P_1_R protein is localized to myocardial and endocardial cells in the developing heart**. Wild type C57Bl/6 embryo sections were immunostained with (A, C, E) control normal rabbit IgG or (B, D, F) a rabbit polyclonal antibody against S1P_1_R. A secondary antibody conjugated to hydrogen peroxidase was used in conjunction with a Vector SG substrate color reaction kit to produce a blue-grey precipitate wherever S1P_1_R was expressed. Sections were counterstained with nuclear fast red to label the nuclei in each cell. S1P_1_R expression was observed in the myocardium (M) of both the atrium (At) and ventricle (V) as well as in endothelial cells (arrows) in both (A, B) E10.5 and (C, D) E12.5 hearts. (E, F) E10.5 embryonic sections, at the level of the somites, depicting vascular endothelial cells (arrow heads) stained with anti-S1P_1_R antibodies. (A, B, C, D) Scale bar = 100 μm, (A and B inserts and E, F) scale bar = 50 μm.

To localize the expression of S1P_1_R at the cellular level, we performed immunohistochemical analysis with an antibody against S1P_1_R. S1P_1_R protein was localized to cardiomyocytes and endothelial cells in the heart at E10.5 and E12.5 (Figure 2). Myocardium of both the ventricle and atrium expressed S1P_1_R protein at E10.5 and E12.5 (Figure 2). In addition, S1P_1_R protein was detected in vascular endothelial cells throughout the embryo (Figure 2). These patterns of S1P_1_R expression are similar to that observed in the adult [8]. These data show that S1P_1_Rs are the primary receptor subtype expressed in the early developing murine heart.

As detailed above, we demonstrate that S1P_1_R is highly expressed from E9.5 to E12.5, stages that span an important developmental period for the heart. During these stages, there is expansion of the cardiac cushions and the beginning of their refinement into cardiac valves [29]. In addition, the myocardium undergoes differentiation and development of the compact outer layer of the heart as well as formation of trabeculae [30]. Our gene expression results were consistent with *in situ *hybridization analysis [11], which demonstrated that of the five S1P receptor subtypes, only S1P_1_R was detected in the developing heart at the stages we examined. Here, we expand on earlier analysis and demonstrate that S1P_1_Rs are expressed at the protein level in both myocardial and endocardial cells during early heart development. Taken together these data indicate that S1P_1_R is expressed at the right time and in the right cells to influence multiple aspects of cardiac development.

### S1P_1_R mediates effects of S1P treatment on AV canal cultures

To identify the S1P receptor subtype(s) that mediate S1P action, agonists and antagonists to specific S1P receptors were applied to AV canals grown in culture. The doses used were previously validated to either block or activate specific S1P receptors [31-33].

**Figure 3:**
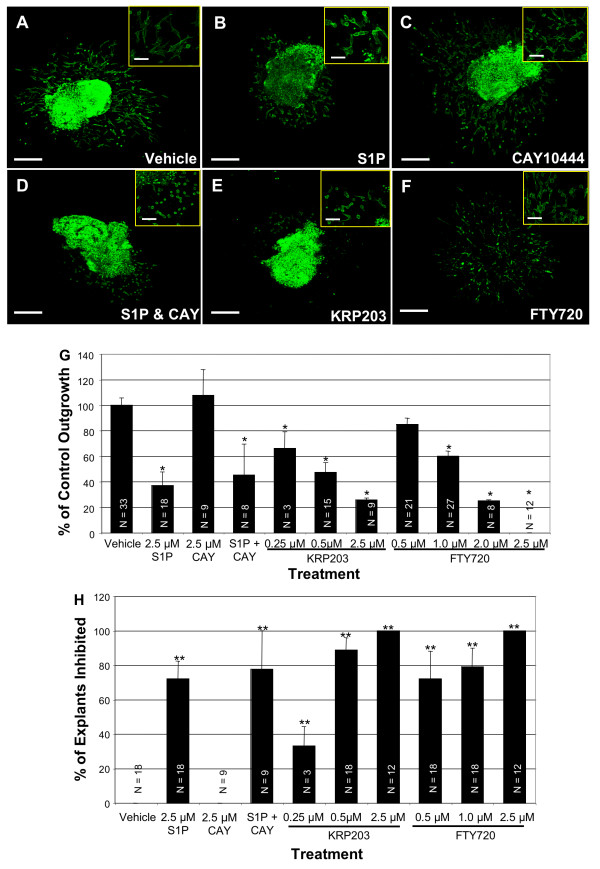
**S1P_1_R mediates cell rounding, cellular outgrowth, and EMT in AV canal cultures**. AV canals were explanted at E9.5 and treated at 2 and 24 hours with vehicle or S1P receptor agonists or antagonists. Explants were stained with phalloidin conjugated to Alexa Fluor 488. (A) Vehicle treated explants show normal cellular outgrowth and elongated cell morphology. (B) S1P (2.5 μM) treatment causes reduced outgrowth and cell rounding. (C) CAY10444 (2.5 μM), an S1P_3_R antagonist, treated cultures have normal cellular outgrowth and cell morphology. (D) CAY10444 (2.5 μM) is unable to rescue explants from S1P (2.5 μM) treatment, as these cultures have inhibited cell migration and rounded cells. (E) The S1P_1_R specific agonist, KRP203 (0.5 μM) causes reduced cellular outgrowth and cell rounding similar to S1P treatment. (F) FTY720 (1.0 μM), an S1P_1,3,4,5_R agonist also causes reduced cellular outgrowth and cell rounding similar to S1P. (G) The average cellular outgrowth for each treatment was calculated and displayed as a percent of the vehicle control cellular outgrowth. (H) Each explant was examined for transformed mesenchymal cells that had invaded into the collagen gel. An explant was scored as inhibited if less than 5 mesenchymal cells had invaded. Results are displayed as a percent of explants that were inhibited. Vehicle control and CAY10444 showed no inhibition, while the other treatments had significant inhibition of mesenchymal cell formation. Scale bar low magnification equals 200 μm, and in high magnification inserts it equals 50 μm. N equals number of explants measured. *P ≤ 6.3 × 10^-5^, **P ≤ 0.0005.

FTY720, an agonist for S1P_1,3,4,5 _when converted to FTY720-P by SPHK2, caused similar effects on AV canal cultures as S1P in a dose-dependent manner (Figure 3). Treatment of AV canals with 0.5 μM FTY720 inhibited cellular outgrowth from the explants by 15%, and inhibited EMT by 72%. Treatment with 1.0 μM FTY720 caused a 40% decrease in cellular outgrowth and a 79% inhibition of EMT (Figure 3). These two doses of FTY720 induced changes in cell morphology, including cell rounding and actin cortical stress fiber formation (Figure 3). This cellular phenotype was in contrast to the fusiform shape of vehicle-treated cells that have many cellular processes extending from the cell body, indicating cellular migration over the surface of the collagen gel (Figure 3). At the highest dose of 2.5 μM, FTY720 completely inhibited cell migration and EMT (Figure 3). In addition, 2.5 μM FTY720 treatment caused explant death, as measured by beating. Only 42% (N = 12) of 2.5 μM FTY720-treated explants were still beating and alive at the end of 48 hours of incubation, compared to 100% for vehicle (N = 39), 0.5 μM FTY720 (N = 9), or 1.0 μM FTY720 (N = 33) treated explants.

We next tested the S1P_1_R-specific agonist KRP203 [34]. KRP203 inhibited cellular outgrowth and EMT in a dose-dependent manner. Treatment with 0.25 μM KRP203 inhibited outgrowth from the AV canal explants by 33.9% and blocked EMT by 33.3% (Figure 3). 0.5 μM KRP203 inhibited cellular outgrowth by 52.6% and inhibited EMT by 88.8% in AV canal cultures (Figure 3). KRP203 caused changes in cell morphology, similar to S1P and FTY720 treatment, which included cell rounding, fewer filapodia extending from the cells, and the formation of actin stress fibers (Figure 3). At a higher dose of 2.5 μM, KRP203 inhibited outgrowth by 74.2% and completely blocked EMT (Figure 3). As with the highest dose of FTY720, 2.5 μM KRP203 caused AV canal explant death. Only 33.3% (N = 8) of 2.5 μM KRP203-treated explants were alive and beating after 48 hours as compared to 100% of explants treated either with vehicle (N = 21) or 0.5 μM KRP203 (N = 12).

**Figure 4:**
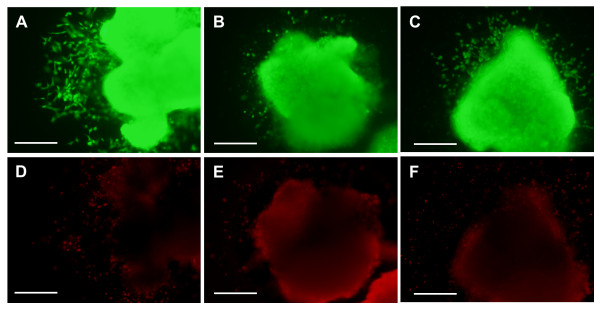
**S1P receptor agonists do not increase cell death in AV canal cultures**. Cell viability was assessed following S1P_1_R receptor agonist treatment. (A, D) AV canal explant treated with vehicle control (methanol). (B, E) explants treated with 0.5 μM KRP203. (C, F) explants treated with 1.0 μM FTY720. (A, B, C) only live cells are labeled with calcein AM and appear green, where as (D, E, F) only dead cells are labeled with ethidium homodimer-1 and appear red. Dead cells are seen around the edges of the explants and in the muscle explants but the majorities of cells fluoresce green and are alive under all treatments. Scale bar = 200 μm.

Since higher doses of FTY720 and KRP203 caused explant death, we tested whether the lower doses of FTY720 (1.0 μM) and KRP203 (0.5 μM) caused an increase in cell death in either endothelial cells or cardiomyocytes of AV canal explants, without causing complete explant death. Using a cell viability assay, we determined that S1PR agonist treatment affects cell morphology and cell migration independent of cell toxicity. The calcein AM (green, live cells) staining clearly marked the majority of the muscle cells in the explant and numerous endothelial cells on the collagen gel surface, regardless of treatment (Figure 4). The ethidium homodimer-1 (red, dead cells) staining showed minimal dead cells in the muscle explant and some dead cells on the surface of the gel with each treatment (Figure 4). However, it did not appear that either FTY720 (1.0 μM) or KRP203 (0.5 μM) treatment increased cell death significantly above what was observed in vehicle-treated cultures (Figure 4).

Another S1P receptor expressed in adult cardiac tissue is S1P_3_R [35]. Although the level of S1P_3_R expression in the developing heart was much lower than S1P_1_R in our real-time PCR experiments, we tested whether it could mediate S1P effects in our AV canal cultures. We treated cultures with CAY10444, a specific S1P_3_R antagonist [33]. CAY10444 (2.5 μM) treatment alone had no adverse effects on the AV canal cultures (Figure 3). Pretreatment of AV canal cultures with CAY10444 was unable to prevent cell rounding or inhibition of cellular outgrowth caused by S1P treatment (Figure 3).

As we reported, S1P action can directly effect cardiac development and differentiation in an *in vitro *culture system [10]. In our current studies, we used agonists and antagonists to specific S1P receptors to determine which receptor subtype mediates S1P action in the developing heart. The S1P_1_R specific agonist (KRP 203) had similar effects on cell morphology and migration as S1P, which indicates that S1P_1_Rs are the critical S1P receptors in the embryonic heart. FTY720 does not bind S1P_2_Rs [31,36], thus we can eliminate S1P_2_Rs as the predominate mediators of S1P signaling in AV canals because FTY720 treatment leads to similar changes in cell motility and morphology as S1P. S1P_3_Rs do not appear to be the primary mediators of S1P action either since an S1P_3_R-specific antagonist could not prevent S1P from inhibiting cell migration and EMT. S1P_4_R is only minimally expressed in the heart as detected by RT-PCR, and S1P_5_R is not at all expressed in the heart, as previously reported [10], both of which suggest that they are not critical for S1P action in the AV canals. Taken together, these data indicate that S1P_1_Rs are the primary mediators of S1P action in cardiac cushion tissue. These experiments, however, do not eliminate the possibility that other receptors like S1P_2_R and S1P_3_R, both of which have a low level of expression in the embryonic heart, could also mediate S1P signaling in the developing heart, especially if S1P_1_R expression is lost. The notion that S1P receptor subtypes can compensate for the loss of other subtypes in the cardiovascular system is supported by data showing that gene knockout of multiple S1P receptor subtypes leads to more severe phenotypes and younger embryonic lethality [20].

### Loss of S1P_1_R expression disrupts cardiac development

To complement pharmacological studies assessing the role of S1P_1_R during heart development, we examined the morphology of S1P_1_R-/- embryonic hearts at two embryonic stages E10.5 and E12.5. Examination of E10.5 hearts was chosen for two reasons. First, cardiac cushion development is well underway at this stage and we could thus compare *in vivo *cushion development with that observed in our *in vitro *AV canal culture system. Second, E10.5 embryos do not show signs of hemorrhage. E12.5 was selected because S1P_1_R-/- embryos are alive at this age and are represented at the correct Mendelian ratio, where as S1P_1_R-/- embryos begin to die at E13.5. Although E12.5 S1P_1_R-/- embryos have some intra-embryonic bleeding and their limbs are underdeveloped, their overall size is similar to S1P_1_R+/+ littermates [20].

**Figure 5:**
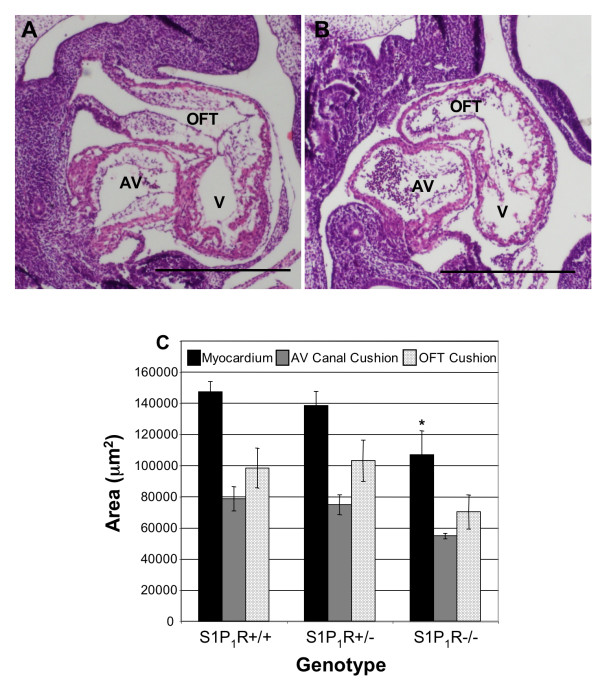
**Loss of S1P_1_R inhibits cardiac growth and morphology at E10.5**. E10.5 embryos were fixed, sectioned saggitally and stained with H&E. (A) S1P_1_R+/+ hearts exhibits normal morphology. (B) S1P_1_R-/- hearts demonstrate reduced myocardial tissue with a thin ventricular wall. (C) The average area of the myocardium at the level of the cardiac cushion, as well as the average area of the atrioventricular (AV) canal cushions and outflow tract (OFT) cushions, were determined. V = ventricle. S1P_1_R+/+, N = 4; S1P_1_R+/-, N = 5; S1P_1_R-/-, N = 3, *P ≤ 0.02. Scale bar equals 500 μm.

E10.5 S1P_1_R-/- embryos were not significantly smaller than S1P_1_R+/+ or S1P_1_R+/- littermates. The crown-rump (CR) lengths of E10.5 embryos were S1P_1_R+/+ 4.63 mm ± 0.38 mm, N = 5; S1P_1_R+/- 4.82 ± 0.22 mm, N = 12; S1P_1_R-/- 4.25 mm ± 0.33 mm, N = 7. However, the hearts of S1P_1_R-/- embryos had reduced myocardial tissue, with ventricular myocardial area reduced by 27% compared to S1P_1_R+/+ littermates (Figure 5). The heart walls in the S1P_1_R-/- primitive ventricle were thinner and the trabeculae were less developed compared to S1P_1_R+/+ and S1P_1_R+/- littermates (Figure 5). No differences in the sizes of the outflow tract (OFT) or AV canal cushions were observed at E10.5 (Figure 5).

**Figure 6:**
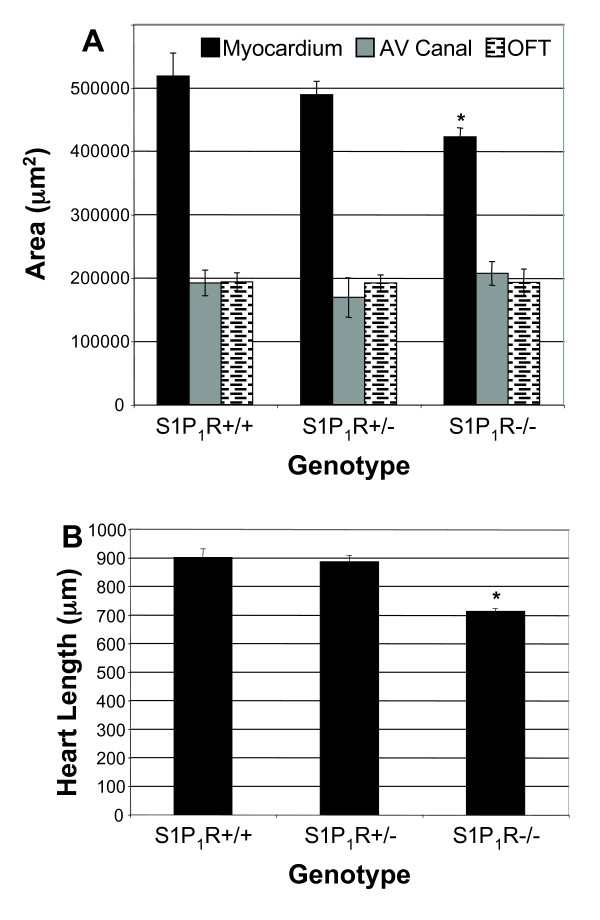
**Loss of S1P_1_R inhibits cardiac growth at E12.5**. (A) The average myocardial area at the level of cardiac cushions and the cardiac cushions were measured. S1P_1_R-/- hearts had less ventricular tissue but were normal for cardiac cushion size, both AV canal and outflow tract. S1P_1_R+/+, N = 5; S1P_1_R+/-, N = 3; S1P_1_R-/-, N = 4. *P ≤ 0.02. (B) The length of the heart was measured from the AV canal cushion to the apex of the heart. S1P_1_R-/- hearts were shorter than S1P_1_R+/+ and S1P_1_R+/- littermates. S1P_1_R+/+, N = 3; S1P_1_R+/-, N = 3; S1P_1_R-/-, N = 3. *P ≤ 0.0006.

**Figure 7:**
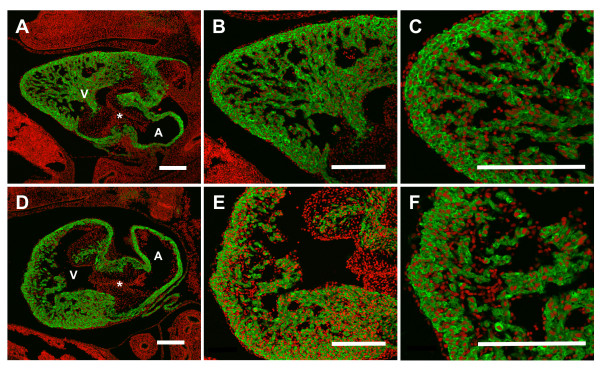
**Loss of S1P_1_R causes altered cardiac morphology at E12.5**. Saggital heart sections were immunostained for sarcomeric α-actin (green) and counterstained with propidium iodide (red) to mark the nuclei. (A, B, C) E12.5 S1P_1_R+/+ heart exhibits normal morphology. (D, E, F) E12.5 S1P_1_R-/- heart demonstrates disrupted morphology, including rounded apex, reduced ventricular tissue and disorganized compact layer and trabeculae. (A, D) 100X, (B, E) 200X, and (C, F) 400X magnification. Scale Bar = 200 μm. V = ventricle, A = atrium, * = AV canal cushion.

No differences in embryo sizes were observed between S1P_1_R genotypes at E12.5. The CR lengths of E12.5 embryos were S1P_1_R+/+ 8.54 mm ± 0.16 mm, N = 6; S1P_1_R+/- 8.64 mm ± 0.09 mm, N = 19; S1P_1_R-/- 8.38 mm ± 0.25 mm, N = 10. S1P_1_R-/- hearts, though, were smaller with a shortened long axis and reduced overall ventricular tissue (Figures. 6, 7). The average length of S1P_1_R-/- hearts was 21% less than S1P_1_R+/+ hearts when measured from the AV canal to the apex of the heart (Figure 6). S1P_1_R-/- hearts had 21% less ventricular myocardial tissue than S1P_1_R+/+ hearts (Figure 6). Cardiac cushions in E12.5 hearts did not show differences in size among the different S1P_1_R genotypes (Figure 6).

To assess the myocardial structure of S1P_1_R-/- hearts, immunohistochemistry with antibodies against the myocardial marker sarcomeric α-actin was performed. This analysis revealed disrupted morphology in E12.5 S1P_1_R-/- hearts, they displayed a thickened and more disorganized ventricular myocardial wall and trabeculae compared to the tightly compacted ventricular wall and well-organized trabeculae of S1P_1_R+/+ hearts (Figure 7). In addition, the apexes of the S1P_1_R-/- hearts were blunted and rounded as compared to S1P_1_R+/+ hearts, which exhibited a V shape that tapers to the apex (Figure 7).

**Figure 8:**
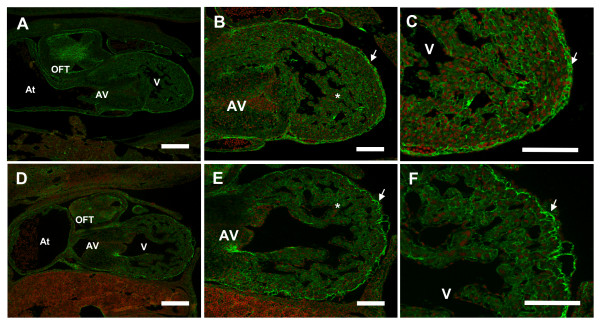
**Reduced fibronectin deposition observed in S1P_1_R-/- hearts**. E12.5 transverse embryo sections were immunostained with an anti-fibronectin antibody followed by a secondary antibody conjugated to Alexa Fluor 488 (green), and counterstained with propidium iodide (red). (A, B, C) S1P_1_R+/+ hearts demonstrate strong FN staining in the outflow tract (OFT) and atrioventricular canal (AV) cardiac cushions, epicardial layer (arrow), and throughout the ventricular (V) myocardium and trabeculae (*). (D, E, F) FN expression in the epicardial layer (arrow) of S1P_1_R-/- hearts revealed a more disorganized layer compared to S1P_1_R+/+ hearts. In addition, the amount of FN deposited surrounding myocardial and trabecular cells was reduced compared to S1P_1_R+/+ hearts. At = atrium. A, D scale bar = 200 μm; B, C, E, F scale bar = 100 μm.

To characterize the cardiac cushion tissue and extra-cellular matrix (ECM) structure in S1P_1_R-/- hearts, we performed immunohistochemistry against fibronectin (FN), an important ECM component in the heart during development. This analysis identified disruptions in the pattern of fibronectin protein expression in the heart at E12.5 (Figure 8). In S1P_1_R+/+ hearts, there was strong staining for FN in the subepicardial layer, discrete FN deposition in the myocardium, and strong FN deposition in the cardiac cushions (Figure 8). In contrast, S1P_1_R-/- hearts showed reduced FN expression throughout the myocardium of the ventricular wall and trabeculae, as well as reduced FN deposition in the cardiac cushions (Figure 8). Although S1P_1_R-/- hearts displayed FN expression in the epicardial layer, this expression revealed a less cohesive epicardial layer with areas detached from the underlying myocardial layer (Figure 8).

To determine if the reduced size of S1P_1_R-/- hearts was due to increased cell death or decreased cell proliferation, we performed TUNEL analysis or immunostained for the proliferation marker phospho-histone H3. We observed a very low rate of cell death in E12.5 hearts of all S1P_1_R genotypes examined. Throughout the entire heart, including the ventricle, atrium and cardiac cushions, there were no more than 3 TUNEL-positive cells per section, and often there were no TUNEL-positive cells in a particular heart section. To quantitate the amount of cell death, myocardial cells were analyzed, as they were the most numerous cell type. The rate of cell death as determined by TUNEL analysis in the myocardium was not significantly different among the three S1P_1_R genotypes. The cell death rates were S1P_1_R+/+ 0.07% ± 0.04, N = 3; S1P_1_R+/- 0.07% ± 0.04, N = 3; S1P_1_R-/- 0.16% ± 0.09, N = 3; (P >0.05, one-way ANOVA). In contrast to cell death, a decrease in the cell proliferation rate was observed in the myocardial cells of E12.5 hearts. S1P_1_R-/- myocardial cells had a reduction in cell proliferation of 33%, the rates were S1P_1_R+/- 0.95% ± 0.05, N = 3, and S1P_1_R-/- 0.63% ± 0.08, N = 3 (P <= 0.02, two-tailed students t-Test).

Examination of S1P_1_R-/- embryonic hearts revealed two distinct problems associated with the loss of S1P_1_R expression. First, S1P_1_R-/- hearts were disproportionately smaller than the rest of the embryo. Second, the overall morphology of the hearts was disrupted at each embryonic stage examined. The decrease in cardiac size is in part due to the reduction in cell proliferation in myocardial cells and not an increase in cell death. S1P has been shown to be a potent stimulator of cell proliferation, especially in cardiovascular cells [8].

The explanation for the disrupted cardiac morphology is less clear. At E10.5 the myocardial layer was thinner in S1P_1_R-/- hearts compared to S1P_1_R+/+ littermates. At E12.5, S1P_1_R-/- hearts displayed an irregular outer myocardial layer of uneven thickness, possibly due to altered cell proliferation rates observed in S1P_1_R-/- hearts.

Another possible explanation for the malformed myocardium in S1P_1_R-/- hearts could be a disruption in the epicardium. Similar defects in cardiac development have been observed when epicardium formation was defective, resulting in thin ventricular myocardium, disrupted compact layer formation, and malformations in cardiac cushion development [37]. An important component of the epicardial layer is the extra-cellular matrix molecule fibronectin, which is critical for the migration of epicardial cells over the myocardium [38]. In addition, it was reported that S1P stimulates FN matrix assembly and that inhibition of sphingosine kinase 1 leads to decreased FN expression [39,40]. This idea that altered S1P signaling disrupts FN expression and leads to cardiac malformations is supported by our immunohistochemical analysis of FN. We observed reduced levels of FN in the cardiac cushions and throughout the myocardium of S1P_1_R-/- hearts. In addition, our analysis of FN revealed a disruption in the epicardial layer, including portions of the layer that have delaminated from the underlying myocardium. This disrupted epicardial layer may explain the disorganized myocardium observed in the compact layer of S1P_1_R-/- hearts; however, further analysis of the role of S1P during epicardial development will be needed to investigate this possibility.

Other than reduced FN expression, there are no major differences in the cardiac cushions of S1P_1_R-/- and S1P_1_R+/+hearts. It is difficult to directly compare the *in vitro *and *in vivo *cardiac cushion results because the alterations in S1P signaling are different. *In vitro*, the loss of S1P signaling, due to a block in S1P production, results in cell death, whereas only S1P_1_R signaling is lost in the *in vivo *transgenic model, which results in reduced cell proliferation. In the *in vivo *model, it is possible that other S1P receptors expressed in the heart may compensate for the loss of S1P_1_R, which is not possible in the *in vitro *model since all S1P signaling is blocked. As for the effects of S1P and S1P_1_R agonists, their treatment causes increased S1P signaling, which is opposite to the *in vivo *model where S1P_1_R signaling is lost. These opposite effects on S1P signaling may explain why the *in vitro *system shows a dramatic effect on cell morphology and migration, where as no gross morphological differences in the cardiac cushions are observed with the *in vivo *model.

S1P_1_R-/- embryos die at E14.5 due to a failure in the maturation of the vasculature, which leads to hemorrhaging and death [15]. These previously reported data support the idea that effects on cardiac development observed in the S1P_1_R-/- embryos could be secondary to vascular defects. However, data in this report indicates that loss of S1P_1_R-/- expression leads to direct effects on cardiac growth and morphogenesis at these early stages, specifically: 1) S1P_1_Rs are highly expressed in the heart, 2) altered S1P signaling in isolated heart tissue causes cell morphology and cell migration defects, 3) S1P_1_R-/- embryos are alive and not smaller than S1P_1_R+/+ littermates, 4) S1P_1_R-/- hearts are smaller than S1P_1_R+/+ littermates, 5) S1P_1_R-/- myocardial cells have reduced cell proliferation rates compared to S1P_1_R+/- myocardial cells *in vivo*. 6) S1P_1_R-/- hearts have reduced FN deposition in the heart compared to S1P_1_R+/+ littermates.

## Conclusions

Our findings identify S1P_1_Rs as the predominate mediators of S1P action in developing AV canal tissue and demonstrate that loss of S1P_1_Rs disrupt normal cardiac development. These findings identify S1P acting via S1P_1_Rs as an important mediator of cardiac development through the regulation of cell morphology, cell proliferation and fibronectin expression. Identifying factors that influence cardiac development is critical for increasing our understanding of the pathogenesis of congenital heart disease, with the ultimate goal of decreasing disease morbidity and mortality. Further studies are required to determine whether factors that alter S1P levels during embryonic development can also influence cardiac development.

## Methods

### Animals

All experiments conducted on animals for this report were first approved by the Institutional Animal Care and Use Committee (IACUC) of Yale University. C57Bl/6 mice were obtained from Charles River Laboratories (Wilmington, MA). The S1P_1_R knockout mouse line was obtained from Dr. Proia at National Institute of Diabetes and Digestive and Kidney Diseases, National Institutes of Health, and has been described [15,20]. All S1P_1_R knockout mice and embryos were genotyped by PCR analysis of genomic DNA isolated from tail tips or yolk sac tissue. Genomic DNA was isolated with a DNeasy Tissue Kit (Qiagen, Valencia, CA), and PCR was performed, as described [15,20]. S1P_1_R+/- vs. S1P_1_R+/- mice were mated so that all genotypes (S1P_1_R+/+, S1P_1_R+/-, and S1P_1_R-/-) were produced. Timed matings were used to obtain the appropriate staged embryos. E0.5 was designated as the day a vaginal plug was observed.

### Chemicals

Sphingosine-1-phosphate (S1P; Sigma-Aldrich, St. Louis, MO) was dissolved in 100% methanol to 5.25 mM and diluted to 250 μM in 0.1% fatty acid-free bovine serum albumin (FAF-BSA; Sigma). FTY720, KRP203, and CAY10444 (Cayman Chemical, Ann Arbor, MI) were resuspended in dimethyl formamide and diluted with 0.1% FAF-BSA.

### Real-Time PCR

E9.5 and E12.5 hearts and embryos were collected under a dissecting microscope, as described [10]. Tissue was rinsed in PBS, placed in a 1.5 ml microcentrifuge tube, flash frozen in liquid nitrogen, and stored at -80°C until RNA isolation. RNA isolation of both hearts (10-12 hearts were pooled per isolation) and embryos without hearts (single embryo per isolation) was performed with the RNeasy Plus Kit (Qiagen) as described [10]. Three separate pools of hearts and three separate embryos with out hearts were used for a biological N of 3. The experiments were carried out in the investigators lab. RNA was eluted in 50 μl of RNase-free water. 1 μg of RNA was reversed transcribed as reported [10]. Each real-time PCR reaction contains IQ SYBR Green Super Mix (Bio-Rad, Hercules, CA), 50 ng cDNA, and 0.5 μM of each primer in a 20 μl reaction volume. PCR was performed at 55°C for annealing with an Opticon 2 DNA Engine PCR machine (Bio-Rad). All primers were designed against mouse sequence including β-actin as a control gene (forward 5'-TGTTTGAGACCTTCAACACC-3', reverse 5'-TAGGAGCCAGAGCAGTAATC-3'). Murine S1P receptor primers for real-time PCR were obtained from SA Bioscience (Frederick, MD). Each real-time PCR experiment was performed in triplicate for each of the biological samples of RNA collected. PCRs were analyzed by mean normalized expression, with β-actin as the control gene. DNA contamination was assessed by a no RT control.

### AV Canal Cultures

AV canal cultures were prepared from E9.5 hearts from C57BL/6 mice, as reported [10,41]. Each culture contained three AV canal explants that were treated with drugs at 2 and 24 hours in culture, and stopped after 48 hours, as described [10]. After 48 hours, AV canal cultures were photographed by phase contrast microscopy using an Olympus IX70 inverted microscope. Images were used to measure cellular outgrowth from the explants with computer software, as described [10]. Mesenchymal cells in the collagen gels were counted for each explant, as reported [10].

Explants were fixed in 4% paraformaldehyde (PFA) and prepared for immunostaining as described [10]. The actin cytoskeleton was examined with phalloidin conjugated to Alexa Fluor 488 (Molecular Probes, Eugene, Oregon) and counterstained with propidium iodide to highlight the nuclei, as described [10].

The level of apoptosis in AV canal cultures was assessed 48 hours after drug treatment with the LIVE/DEAD Viability/Ctotoxicity kit (Molecular Probes), according to the manufacturer's instructions and as reported [10].

### Embryo and Cardiac Morphological Analysis

Gross morphology of the embryos was examined by light microscopy and digital images were captured. Gross abnormalities were recorded upon collection of the embryos and crown-rump lengths were determined from the digital images. Embryos were fixed in 4% PFA overnight at 4°C. Embryos were embedded in paraffin as described [42], sectioned, and stained with hematoxylin and eosin (H&E)[10]. H&E stained tissue sections were used to assess cardiac cushion area, as described [43]. Cardiac cushion ECM was evaluated by immunostaining with antibodies against fibronectin as detailed [42]. H&E sections were used to assess myocardial area and wall thickness, as described [43]. The axial length of the heart from the AV canal cushion to the apex of the heart was determined using Image-Pro Plus software (Media Cybernetics, Silver Springs, MD). Paraffin sections were immunostained with antisera to sarcomeric α-actin (Sigma-Aldrich), as described [42], in order to highlight myocardial wall structure, trabeculation, and septation. Sarcomeric α-actin staining was examined with an Olympus Fluoview laser scanning confocal microscope, as reported [10].

Paraffin sections, of E12.5 embryos (C57Bl/6) fixed in 4% PFA, were immunostained with antisera to S1P_1_R (EDG-1 (H-60) sc-25489, Santa Cruz Bioechnology Inc., Santa Cruz, CA). The Vectastain Elite ABC Kit (Vector Laboratories, Burlingame, CA) was used in combination with the Vector SG substrate kit (Vector Laboratories) according to the manufacturer's specifications to visualize S1P_1_R protein expression. The resulting color reaction caused a blue-gray precipitate to form where S1P_1_R was expressed. Sections were also counterstained with nuclear fast red (Vector Laboratories) to mark the nuclei of each cell. As a control for S1P_1_R antibody staining, normal rabbit IgG (Santa Cruz) was used at the same concentration as the primary antibody for S1P_1_R, which was 2 μg/ml.

### TUNEL and Cell Proliferation Analysis

Embryonic heart sections at E12.5 were assayed for cell death with the In Situ Cell Death Detection kit (Roche, Mannheim, Germany) according to the manufacturer's instructions and as reported [10,42]. The number of apoptotic and total number of cells were counted in four sections of ventricular myocardium per heart, between 2000 and 3500 cells were counted per heart. Three hearts per S1P_1_R genotype were analyzed. The percentage of TUNEL positive cells divided by the total number of myocardial cells was the rate of cell death.

Cell proliferation in embryonic myocardial cells was determined by immunohistochemical analysis with an anti-phospho-histone H3 (Ser-10) antibody (Millipore Corp., Billerica, MA). Embryos were fixed and sectioned, as described above. In addition to the anti-phospho-histone H3 antibody, an anti-sarcomeric α-actin primary antibody was used to label myocardial cells in the embryonic hearts. After primary antibody incubation, sections were incubated with secondary antibodies, goat anti-rabbit IgG Alexa Fluor 546 (for H3) and goat anti-mouse IgM Alexa Fluor 488 (for actin; Molecular Probes). Nuclei were counterstained with 4',6-Diamidino-2-phenylindole dihydrochloride (DAPI; Sigma). Between 8,000 and 12,000 cells were counted in the ventricular myocardium for each embryonic heart. Counts were taken from at least 10 sections throughout the whole heart. The cell proliferation rate is equal to the number of H3-positive cells divided by the total number of cells counted (DAPI-positive) per heart. Three hearts per S1P_1_R genotype were analyzed.

#### Statistical Analysis

Data are presented as mean +/- SEM. Analyses were performed using Microsoft Excel (Microsoft, Redmond, WA) and GraphPad Prism (GraphPad Software, San Diego, CA). Statistical comparisons between groups were performed by Student *t *Tests (two sample assuming equal variances) and one-way ANOVA with Bonferroni post test. P < 0.05 was considered to indicate statistical significance.

## Authors' contributions

CCW designed experiments, analyzed data and wrote the manuscript. CCM performed and analyzed the real-time PCR experiments and analyzed AV canal data. RRP performed and analyzed histology experiments. SAR assisted in study design and analysis. All authors have read and approve this manuscript.
